# Siponimod supports remyelination in the non-supportive environment

**DOI:** 10.1038/s41598-025-87825-8

**Published:** 2025-02-04

**Authors:** Johann Krüger, Newshan Behrangi, David Schliep, Leo Heinig, Elise Vankriekelsvenne, Nicole Wigger, Markus Kipp

**Affiliations:** Institute of Anatomy, Rostock University Medical Center, Gertrudenstraße 9, 18057 Rostock, Germany

**Keywords:** Multiple sclerosis, Cuprizone, Siponimod, Remyelination, Sphingosine-1-phosphate, Diseases of the nervous system, Myelin biology and repair, Neuroimmunology, Regeneration and repair in the nervous system, Multiple sclerosis, Multiple sclerosis

## Abstract

Inflammatory demyelination, a hallmark of multiple sclerosis (MS) lesions, leads to functional impairments and progressive axonal loss over time. Although remyelination is thought to protect axons, endogenous regenerative processes are often incomplete or fail entirely in many MS patients. While the precise reasons for remyelination failure remain unclear, repeated demyelination in previously affected white matter regions is a recognized contributing factor. In a previous study, we demonstrated that the sphingosine-1-phosphate modulator Siponimod ameliorates metabolic oligodendrocyte injury in an MS animal model. In this study, we explored the potential of Siponimod to enhance remyelination in a non-supportive environment. To this end, male mice were subjected to Cuprizone intoxication for seven weeks. From the onset of the fifth week, when oligodendrocyte progenitor cells begin to differentiate, mice were administered either a vehicle or Siponimod solution. Post-treatment, brain specimens were processed for (immune-) histochemical analyses. After four weeks of Cuprizone intoxication, staining intensities for various myelination markers, were significantly reduced. At the end of week seven, loss of myelin staining intensities was still pronounced, but anti-myelin basic protein (MBP) and myelin-associated glycoprotein (MAG) expression was significantly higher in Siponimod- versus vehicle-treated mice. Consistent with this finding, densities of OLIG2^+^ oligodendrocytes significantly recovered in Siponimod-treated but not in vehicle-treated mice. This enhanced recovery was paralleled by the trend of lower densities of Ki67^+^ proliferating oligodendrocyte progenitor cells. Our findings suggest that Siponimod has modest pro-regenerative capacities, partly explaining the amelioration of disease progression in secondary progressive MS patients.

## Introduction

Multiple sclerosis (MS) is a chronic, neuroinflammatory condition that causes neurological impairments due to complex pathologies in the brain and spinal cord. The initial phase of the disease, known as relapsing–remitting MS (RRMS), is characterized by episodes of evident neurological symptoms (relapses) followed by complete or incomplete recovery (remissions). This phase is mainly driven by focal inflammatory demyelination, peripheral immune cell recruitment, reactive gliosis, and axonal injury. In many patients, the disease advances to secondary progressive multiple sclerosis (SPMS), characterized by a gradual worsening of neurological symptoms independent of relapses^1^. The histopathological features of SPMS are less well understood but are believed to include diffuse white and grey matter microgliosis, subpial demyelination, and transsynaptic degeneration. Approximately 15% of patients experience continuous neurological decline from symptom onset, without initial relapses or remissions, a condition termed primary progressive MS (PPMS).

The precise mechanisms driving progressive neurodegeneration in MS remain elusive, but inadequate remyelination is believed to be pivotal^2–4^. Remyelination is an intricate biological event that, at the individual cellular level, can be delineated into four sequential stages: (I) Oligodendrocyte progenitor cell (OPC) proliferation; (II) OPC migration to demyelinated axons; (III) OPC differentiation; and (IV) engagement of the nascent oligodendrocyte with the axon, known as axonal wrapping^5^. Early neuropathological investigations have recognized that in MS, demyelinated lesions can undergo remyelination. The presence of 'shadow plaques,' indicative of remyelinated lesions, demonstrates the potential for full MS plaque restoration. Nevertheless, partial reparations, primarily at lesion peripheries, are more frequently observed^6,7^. Notably, there is clear evidence that prompt and efficient remyelination protects neurons from degeneration^8^.

Several reports suggest that recurrent demyelination of previously affected white matter contributes to remyelination failure in MS^9–11^. For instance, a detailed histopathological study found that about 15% of remyelinated shadow plaques exhibited new demyelinating activity^9^. Another study using serial magnetization transfer imaging, indicated that new lesions often form in areas of prior lesions undergoing a second round of inflammatory demyelination^11^. Consistent with these findings, several pre-clinical studies have demonstrated recurrent demyelination at sites of previous damage^12–14^. How to protect such brain areas prone to recurrent demyelinating events is currently unknown.

The Cuprizone model serves as a valuable tool to address such questions. In this paradigm, oral administration of the copper-chelator Cuprizone rapidly triggers oligodendrocyte apoptosis and/or ferroptosis, which is closely followed by the activation of the brain’s innate immune cells, astrocytes and microglia, culminating in the demyelination of specific white and grey matter brain regions^15,16^. After 5 weeks of Cuprizone intoxication (called acute demyelination), there is complete demyelination of distinct subregions, paralleled by extensive microglial and astrocytic proliferation and damage to axons^17–20^. If the animals are provided standard chow after acute Cuprizone-induced demyelination, endogenous remyelination occurs, which is complete on the histological level after 3–4 weeks^20^. However, if the Cuprizone intoxication is continued, creating a non-supportive environment with permanent cellular stress, differentiating OPCs die and, as a consequence, remyelination fails.

In a previous study we demonstrated that Siponimod, a modulator of sphingosine-1 phosphate receptors (S1P), ameliorates Cuprizone-induced demyelination^21^. Siponimod selectively binds to two of the five G protein-coupled receptors for S1P: S1P receptor 1 (S1PR1) and S1P receptor 5 (S1PR5). Functionally, Siponimod prevents lymphocytes from exiting the lymph nodes, thereby reducing T-cell recirculation into the central nervous system^22^. This leads to a reduction of inflammatory attacks driven by the peripheral immune system. In this study, we asked whether Siponimod might also protect maturating OPCs thereby allowing remyelination in the non-supportive environment.

## Materials and methods

### Animals, experimental groups and rationale of the experimental design

This work followed the PREPARE (Planning Research and Experimental Procedures on Animals: Recommendations for Excellence) and ARRIVE guidelines (Animal Research: Reporting of In Vivo Experiments)^23^. All procedures concerning the handling of animals, including finalization were performed according to the recommendations of the FELASA (Federation-of-European-Laboratory-Animal-Society-Associations) and approved by the district government’s animal care review boards (Regierung Mecklenburg-Vorpommern, reference number 7221.3-1-001/19).

Male, 6-week-old C57BL/6 mice were purchased from Janvier Labs (Le Genest-Saint-Isle, France). The mice were maintained at a maximum of five animals per cage (100 cm^2^) with ad libitum food and water under standard laboratory conditions (artificial day-night cycle of 12 h each, a room temperature of 22 °C ± 2 °C and a humidity of 50% ± 10%). Cages were changed weekly, and microbiological monitoring was performed according to the recommendations of the FELASA. Animals were allowed to acclimate to the environment for at least one week before disease induction.

**Fig. 1:**
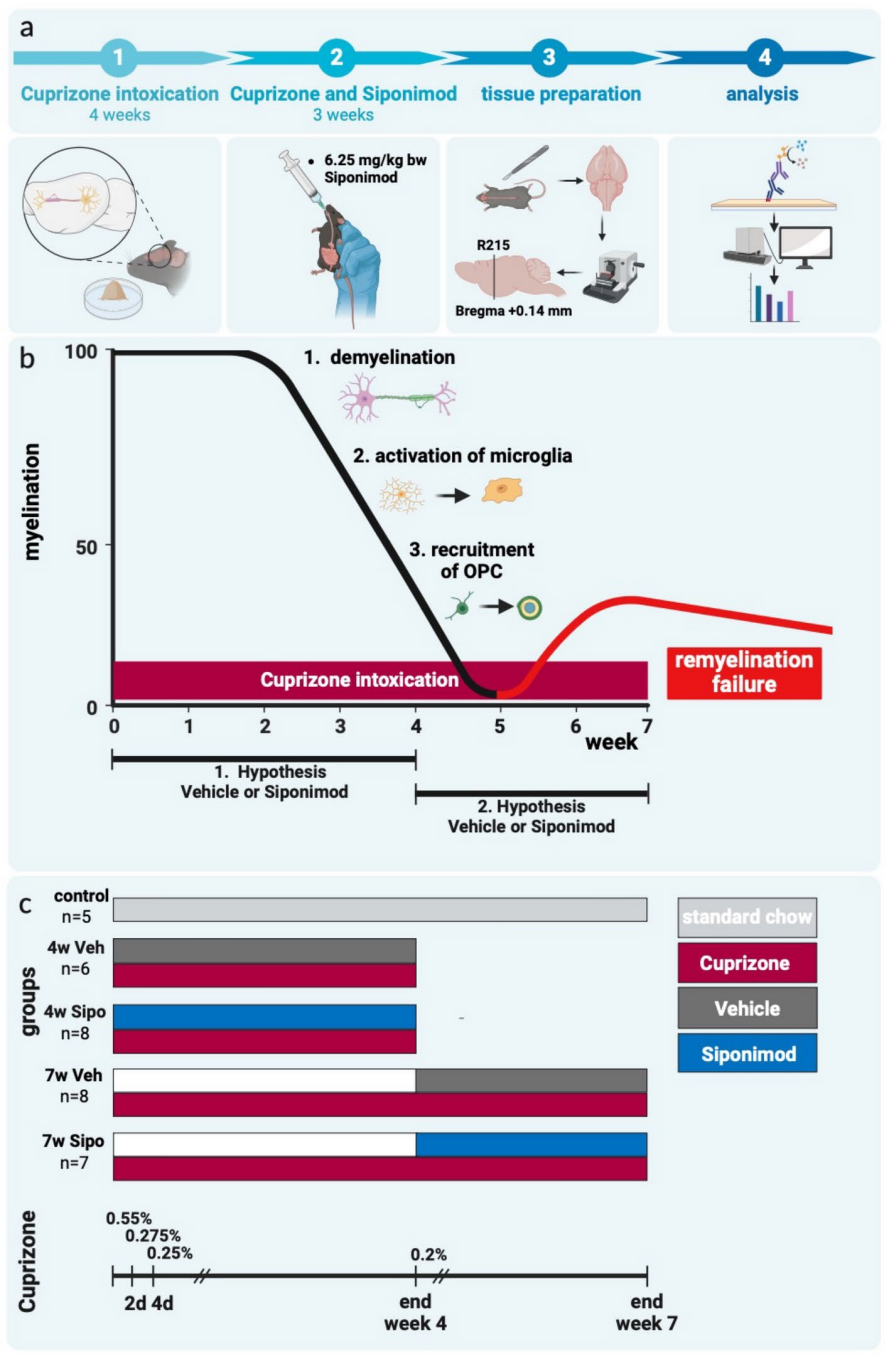
Schematic depiction of the experimental setup. (**a**) General setup of the study. (**b**) Illustration of the cellular characteristics of the Cuprizone model. X-axis numbers indicate the duration of the experiment in weeks. Y-axis numbers indicate an estimate of the percentage of myelinated fibres in the corpus callosum. (**c**) Illustration of the experimental setup. X-axis numbers indicate the duration of the experiment in weeks. Normal chow periods are coloured in light grey, cuprizone intoxication periods in red, vehicle-treatment periods in dark grey and Siponimod-treatment periods in blue, respectively.

Group names consist of the time spent in the experiment plus the type of intervention they received: i.e. 7w Sipo means mice spent seven weeks in the experiment and received Siponimod (for details concerning duration of intervention see Fig. 1c). Upon arrival, mice were randomized into the following study groups: *Control* animals (n = 5) received normal chow for seven weeks. *4w Veh* (n = 8) and *4w Sipo* (n = 8) mice were intoxicated with Cuprizone for four weeks and simultaneously treated with either daily vehicle or Siponimod solution. At the end of week four, the mice were sacrificed, transcardially perfused and processed for (immune-) histochemistry. At this time point, demyelination is ongoing and OPCs are actively recruited to the site of the demyelinating lesion (id est, the corpus callosum; see Fig. 1)^24–27^. *7w Veh* (n = 8) and *7w Sipo* (n = 8) mice were intoxicated with Cuprizone for seven weeks and treated with either daily vehicle or Siponimod solution, starting at the beginning of week five. Between weeks five and seven, the OPCs begin to differentiate into mature, myelinating oligodendrocytes. However, due to the continuous Cuprizone exposure, these cells eventually die and remyelination fails.

### Cuprizone intoxication

Cuprizone intoxication was performed as described previously^28^. In brief, mice were intoxicated with a diet containing Cuprizone (Sigma-Aldrich, Taufkirchen, Germany) mixed into a ground standard rodent chow (V1530–0; Ssniff). To this end, Cuprizone was weighed using precision scales and mechanically mixed with ground standard rodent chow using a commercially available kitchen machine (Kult X, WMF Group, Geislingen an der Steige, Germany). The chow was mixed at low speed and manual agitation for 1 min and provided within the cage in two separate plastic petri dishes. Control animals received standard ground rodent chow throughout the entire study period. A daily health check was conducted throughout the experiment. The mice were assessed based on activity, breathing, movement, fur condition, hydration, and overall constitution. Additionally, their weight was recorded every other day. The following exclusion criteria were applied: severe weight loss (> 10% within 24 h), severe behavioural deficits (decreased locomotion, convulsions, stupor), or infections. No animal met the exclusion criteria during the cuprizone intoxication. One mouse in the *7w Sipo* group was excluded due to a hydrocephalus. Two animals in the *4w Veh group* were excluded due to a proliferative mass in between the two hemispheres. No blinding was performed during Cuprizone-intoxication.

### Drug treatment

Siponimod-treatment was performed via oral gavage as published previously^21^. Siponimod was provided as a powder formulation from Novartis Pharma AG, Basel, Switzerland and dissolved in a 1% carboxymethyl cellulose sodium solution (Sigma-Aldrich; order no. 4888-500gr). Mice were treated daily with a Siponimod concentration of ∼6.25 mg/kg bw (200 µl). Vehicle animals received the same volume of the 1% carboxymethyl cellulose sodium solution at the same time. No blinding was performed during drug treatment.

### Tissue preparation and tissue embedding

For histological and immunohistochemical studies, mice were anesthetized with intraperitoneal ketamine (100 mg/kg) and xylazine (10 mg/kg) injections and then transcardially perfused with 20 ml of ice-cold phosphate-buffered saline (PBS) followed by 50 ml of 3.7% paraformaldehyde solution (PFA; pH 7.4). Histological and immunohistochemical analyses were performed using paraffin-embedded 5 μm-thick coronal brain sections. Analyses were performed at the level 215 according to the mouse brain atlas by Sidman et al. (http://www.hms.harvard.edu/research/brain/atlas.html). Region 215 (R215) corresponds to the stereotaxic coordinates Bregma + 0.14 mm, provided by Franklin and Paxinos^29^.

### Luxol fast blue/periodic acid Schiff stain and evaluation

Previously established protocols were used to perform Luxol Fast Blue/Periodic Acid Schiff (LFB/PAS)-staining^30^. The LFB/PAS-stained sections were differentiated using a 0.05% lithium carbonate solution until the grey matter stained only faintly blue. To evaluate the extent of demyelination in LFB/PAS stains, a nonparametric, blinded grading approach was performed with the midline of the corpus callosum representing the region of interest (ROI). Sections were blindly scored on a scale from 0 (complete demyelination) to 4 (fully myelinated), the results were averaged, and statistically compared.

### Silver staining

Bodian silver staining, modified by Naoumenko and Feigin^31^ was used to visualise axonal fibre densities. After deparaffinisation and washing in distilled water, the sections were incubated in a silver solution with buffered sodium chloride at 56 °C for 24 h. The next day, the sections were reduced using hydroquinone/sodium sulphite solution and further developed in 1% gold chloride solution and 2% oxalic acid at 30 °C. This was followed by fixation in 5% sodium thiosulphate.

### Immunohistochemistry and evaluation

Previously established protocols were used to conduct immunohistochemistry^30^. In brief, sections were deparaffinized, rehydrated, and, if necessary, antigens were unmasked by heating in tris(hydroxymethyl)aminomethane/ethylenediaminetetraacetic acid (Tris/EDTA) buffer (pH 9.0). After washing in PBS, the sections were incubated for 1 h in blocking solution (i.e. 5% normal serum of the species, in which the secondary antibodies were raised). After draining the blocking solution, sections were incubated overnight at 4 °C with the primary antibodies diluted in the blocking solution. A list of antibodies used in this study is provided in Table 1. Appropriate negative controls were performed in parallel, such as omitting primary antibodies. The slides were incubated in a 0.35% hydrogen peroxide (H_2_O_2_) solution in PBS for 30 min the next day. After washing in PBS, the slides were incubated in solution containing horseradish peroxidase-conjugated polymers linked to a secondary antibody (DAKO EnVision™ + System Peroxidase) for 1 h at ambient temperature. The antigenic sites were detected by a reaction with 3,3’-diaminobenzidine (DAKO, Hamburg, Germany) and H_2_O_2_ yielding a brownish deposit.

**Table 1:** Antibodies used for immunohistochemistry and immunofluorescence studies.

Antigen	Application	Host	Utilization	Dilution	Purchase number	RRID	Supplier	Antigen retrieval
APP	IHC	Mouse monoclonal	Primary	1:5000	3293717	AB_94882	Millipore	Tris/EDTA
IBA-1	IHC	Rabbit polyclonal	Primary	1:1000	019-19741	AB_839504	Wako, US	Tris/EDTA
Mouse-IgG	IHC	Goat polyclonal	Secondary	–	K4001	AB_2827819	DAKO	–
Mouse-IgG	IF	Donkey polyclonal	Secondary	1:250	A21203	AB_2535789	AB_2535789	–
Ki67	IF	Rabbit monoclonal	Primary	1:250	MA5-14520	AB_10979488	Thermo Fisher Scientific	Tris/EDTA
MAG	IHC	Mouse monoclonal	Primary	1:4000	Ab89780	AB_2042411	Abcam	Tris/EDTA
MBP	IHC	Rabbit monoclonal	Primary	1:5000	ab218011	AB_2895537	Abcam	–
OLIG-2	IHC	Rabbit polyclonal	Primary	1:500	AB9610	AB_570666	Millipore, US	Tris/EDTA
OLIG-2	IF	Mouse monoclonal	Primary	1:250	MABN50	AB_10807410	Millipore, US	Tris/EDTA
PLP	IHC	Mouse	Primary	1:5000	MCA839G	AB_2237198	Serotec/Biorad	–
Rabbit-IgG	IHC	Goat polyclonal	Secondary	–	K4003	AB_2630375	DAKO	–
Rabbit-IgG	IF	Donkey polyclonal	Secondary	1:250	ab150065	AB_2860569	Abcam	–

*IHC* immunohistochemistry, *IF* immunofluorescence, *RIDD* research resource identifiers.

To analyse staining intensities, images captured with a Leica DM6 B microscope connected to a DMC 6200 camera system (Leica, Wetzlar, Germany), were imported into ImageJ (version 1.52a for Windows, Wayne Rasband, National Institutes of Health, USA). After automated thresholding, the images were binary-converted, and the fraction of dark pixels was automatically analysed. Staining intensity is expressed as a percentage area, with 100% representing maximum and 0% representing minimum staining intensities. The results are shown as staining intensity [%] in the entire ROI (id est, the midline of the corpus callosum).

To analyse OLIG2^+^ cell and APP^+^ spheroid densities in immunohistochemically processed sections, brains were digitalized using a slide scanner (Grundium Ocus®20) and then imported into QuPath (version 0.5.1 for Mac^32^). After manually outlining the ROI and setting a threshold for positive cell detection, automatic detection was performed. Results are averaged and given as cells or spheroids/mm^2^.

### Immunofluorescence labelling and evaluation procedure

Immunofluorescence double-labelling experiments were performed to visualize OLIG2 and Ki67 expression. For immunofluorescence double-labelling, paraffin sections were deparaffinized, rehydrated, and unmasked by heating in Tris/EDTA buffer. Sections were blocked using 5% serum from the same species the secondary antibodies were raised, followed by overnight incubation at 4 °C with two primary antibodies diluted in blocking solution (see Table 1). After washing in PBS, the sections were incubated for 2 h with a combination of fluorescent secondary antibodies, washed again in PBS, and mounted in Fluoroshield™ with DAPI to stain cell nuclei. Negative controls were conducted to exclude nonspecific binding of the fluorescent secondary antibodies. This involved incubating sections with one primary antibody and then with the non-corresponding secondary antibody. Additionally, sections were incubated with fluorescent secondary antibodies alone to further exclude nonspecific bindings. Stained sections were documented using a Leica DM6 B epifluorescence microscope and the Leica Application Suite X software (version 3.7.0.20979, 2019, Germany). For analyses, images were imported into QuPath, and all cells were identified in the DAPI channel using integrated cell detection. Next, OLIG2 and Ki67 expressing cells were classified in the respective channels using individual thresholds for each antigen. The channels were subsequently merged, and the absolute number of detections within the ROI was normalized to the area (cells/mm^2^).

### Statistical analyses

No blinding was performed at the levels of Cuprizone intoxication and drug treatment. After tissue embedding, sectioning, and staining all evaluators were blinded during the described analyses. Data are given as arithmetic means ± standard error of the mean. Differences between the groups were statistically tested using Prism (version 10.1.0, GraphPad Software Inc., San Diego, CA, USA) with confidence intervals of 0.05. The following symbols indicate the significance level: *p ≤ 0.05, **p ≤ 0.01, ***p ≤ 0.001. If not significant, p-values are given on the graph. The acquired data was averaged for every animal, no pseudo-replicates have been included into the performed statistical tests. Due to technical challenges, one control animal was excluded from the analyses for anti-PLP, silver and anti-OLIG2 stains.

In this work, we focused on two distinct hypotheses: Firstly, does Siponimod ameliorate Cuprizone-induced demyelination as previously published by our group^21^? To verify or falsify this first hypothesis, *4w Veh* and *4w Sipo* groups were statistically compared using the non-parametric Mann–Whitney test. Secondly, does Siponimod allow remyelination in a non-supportive environment? To verify or falsify this second hypothesis *7w Veh* and *7w Sipo* groups were statistically compared using the non-parametric Mann–Whitney test. The significance levels for these analyses are shown above the bars in the figures. No outliers were excluded from the statistical tests. Due to the low number of animals in this study, non-parametric tests were used. Furthermore, analysis for normal distribution by Shapiro–Wilk test showed that most of the data was not normally distributed.

## Results

In the first set of experiments, we aimed to verify recent findings that Siponimod protects against Cuprizone-induced demyelination^21^. To this end, mice were intoxicated with Cuprizone for four weeks and, in parallel, treated with either vehicle or Siponimod solution via oral gavage. Figure 1 shows a synopsis of the experimental design (Fig. 1a), the underlying cellular dynamics (Fig. 1b) and the different groups included into the study (Fig. 1c).

**Fig. 2:**
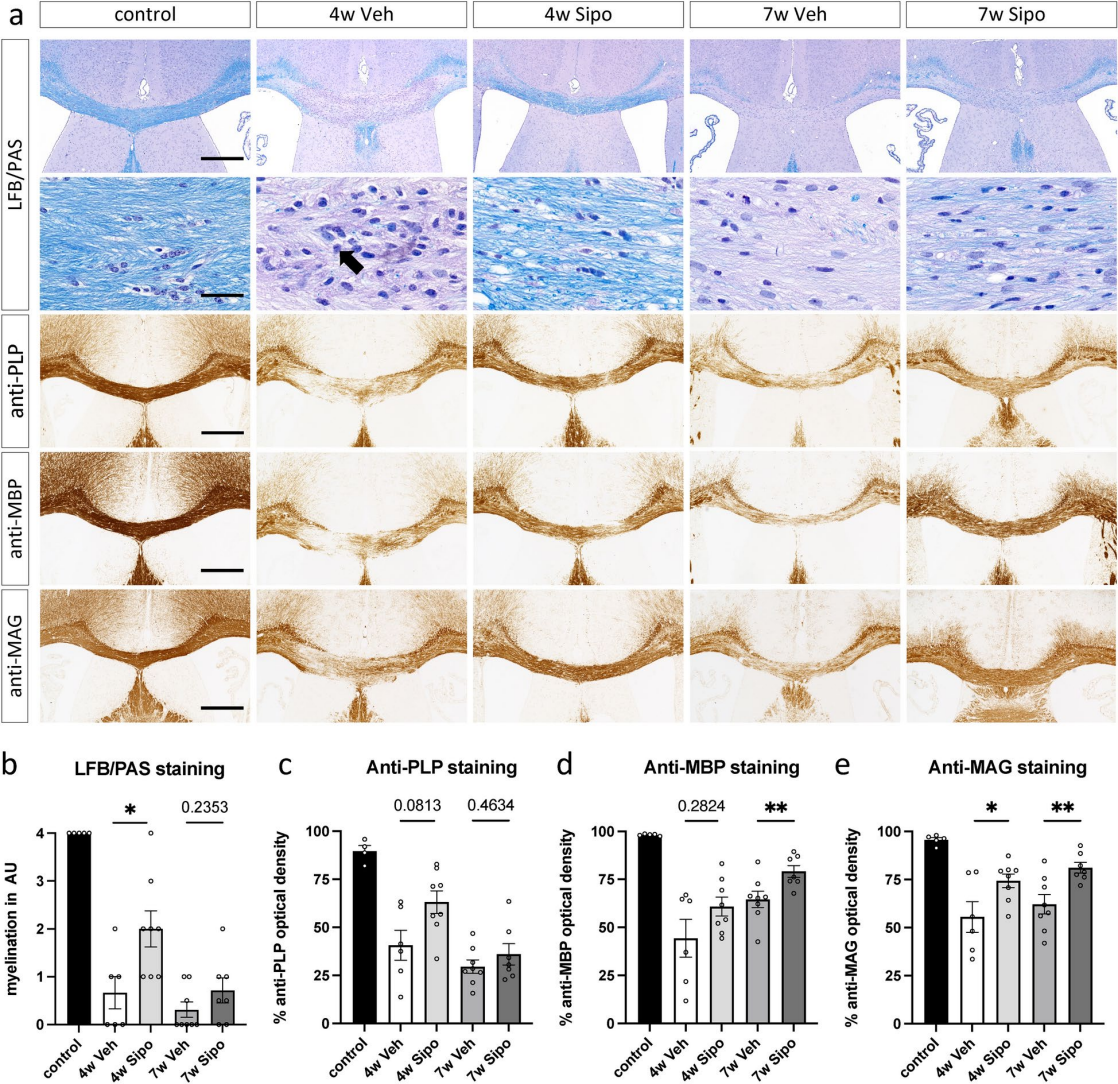
Effect of Siponimod on remyelination in the non-supportive environment. (**a**) Representative images of LFB/PAS, anti-PLP, anti-MBP and anti-MAG-stained sections of the midline corpus callosum at the level of the anterior commissure. Scale bars: 500 μm; 2nd row: 20 µm. The black arrow indicates a cell nucleus. (**b**) LFB/PAS scores in the mCC, assigned from 4 (full myelination) to 0 (no myelination). Densitometric analyses of (**c**) anti-PLP, (**d**) anti-MBP and (**e**) anti-MAG staining intensities (*control* n = 5*, 4W Veh* n = 6, *4W Sipo* n = 8, *7W Veh* n = 8, *7W Sipo* n = 7). One *control* animal was excluded from the PLP analysis due to insufficient staining. Statistical analysis: Mann–Whitney test.

To analyse the extent of demyelination, LFB/PAS (1st and 2nd row in Fig. 1a), anti-PLP (3^rd^ row in Fig. 1a), anti-MBP (4th row in Fig. 1a) and anti-MAG stains (5th row in Fig. 1a) were performed, and the staining intensities were (semi-) quantified in the midline of the corpus callosum at the level of the anterior commissure (id est, R215). As expected, all four stains that were used to visualize myelination levels in the white matter tract corpus callosum revealed a loss of staining intensities, however, to a different extent (see Fig. 2). The loss of LFB/PAS staining intensities was almost complete and paralleled by accumulation of PAS^+^ material and cell nuclei (see arrow in Fig. 2a, 2nd row and Fig. 2b for quantification). In contrast, the loss of anti-PLP (Fig. 2c), anti-MBP (Fig. 2d) and anti-MAG (Fig. 2e) staining intensities was incomplete yet clearly visible. In line with previous observations, this myelination decrease was partially ameliorated in mice daily treated with Siponimod solution (p < 0.05 for LFB/PAS and anti-MAG stains).

To determine the potential effects of Siponimod on intrinsic remyelination capacity during continuous toxin-induced demyelination, mice were intoxicated with Cuprizone for seven weeks. At the onset of week five, both cohorts were treated with either vehicle or Siponimod solution until the end of the experiment (see as well Fig. 1b; 2nd hypothesis for a schematic illustration). After week seven, LFB/PAS staining intensities remained at a low level in the midline of the corpus callosum, but the observed hypercellularity ceased (see 2nd row in Fig. 2a). Animals that began daily Siponimod treatment at the start of week five showed higher myelination levels in all four stains by week seven, compared to those receiving vehicle treatment. This difference was significant for anti-MBP (7w Veh 64.57 *versus* 7w Sipo 79.13; p = 0.0059) and anti-MAG stains (7w Veh 62.09 *versus* 7w Sipo 81.13; p = 0.0093).

**Fig. 3:**
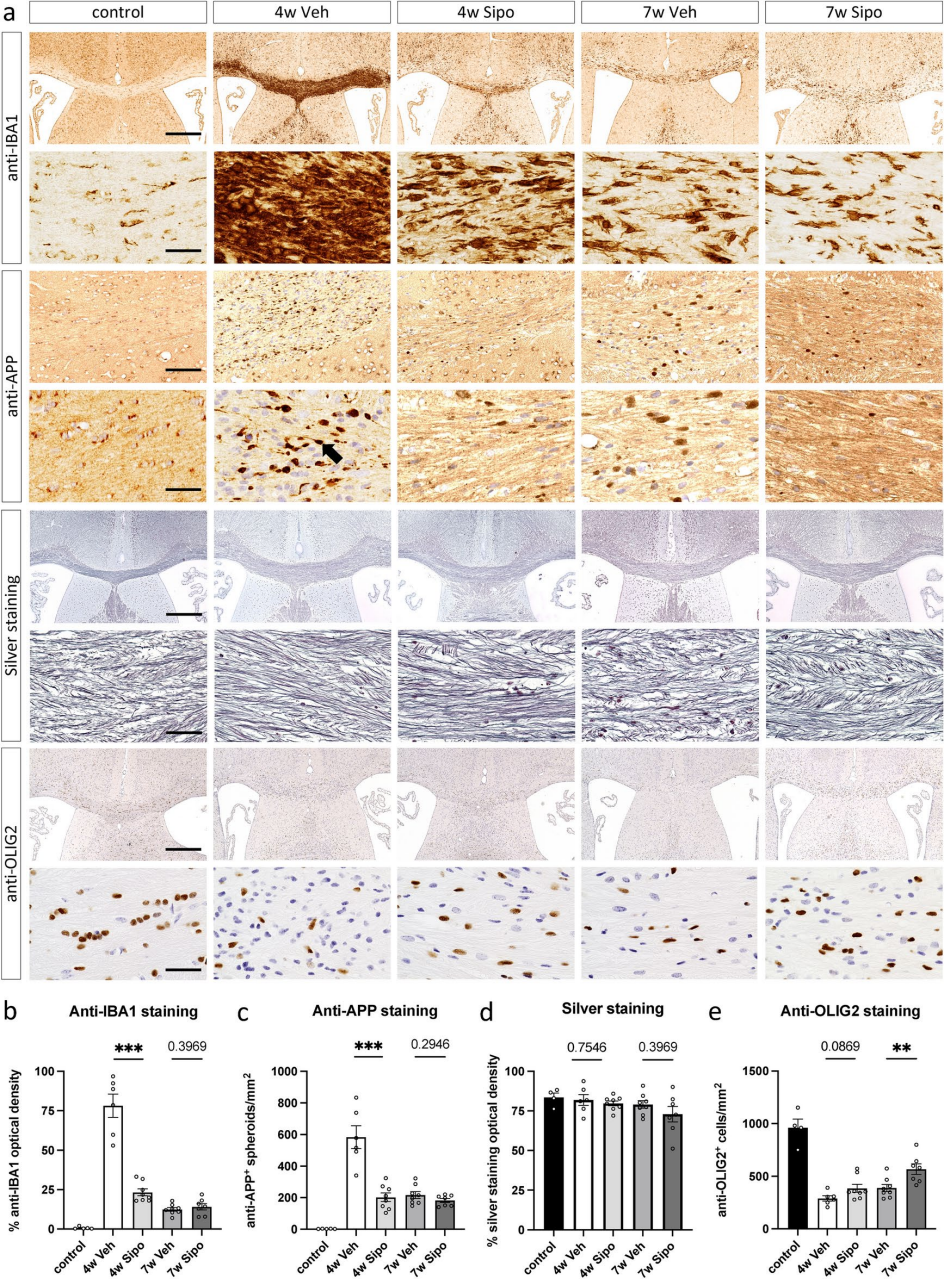
Effects of Siponimod on microgliosis, axonal damage, axonal density and oligodendrocytes. (**a**) Representative images of anti-IBA1 (scale bar upper row 500 and lower row 20 µm), anti-APP (scale bar upper row 100 and lower row 20 µm), silver (scale bar upper row 500 and lower row 20 µm) and anti-OLIG2 (scale bar upper row 500 and lower row 20 µm) stained sections of the midline corpus callosum at the level of the anterior commissure. The black arrow indicates an APP spheroid. (**b**) Densitometric analyses of anti-IBA1, (**c**) particle density analyses of anti-APP, (**d**) densitometric analyses of axonal density and (**e**) cell density analyses of anti-OLIG2 stained sections (*control* n = 5, *4W Veh* n = 6, *4W Sipo* n = 8, *7W Veh* n = 8, *7W Sipo* n = 7). One *control* animal was excluded from the silver and OLIG2 staining analysis due to insufficient staining. Statistical analysis: Mann–Whitney test.

Cuprizone-induced demyelination is paralleled by microglia activation and the axonal accumulation of presynaptic proteins, indicative of deficits in the anterograde axonal transport machinery^33^. In a next step, we were interested to what extent these histopathological hallmarks of the model are altered by Siponimod treatment. Beyond, we investigated the densities of OLIG2^+^ cells, a commonly used pan-oligodendrocytic marker. Figure 3a shows representative images in low and high magnification, whereas Fig. 3b–e show the (semi-) quantitative results. After week four there was a pronounced accumulation of IBA1^+^ cells (Fig. 3b) and APP^+^ axonal spheroids (Fig. 3c). Both histopathological changes were significantly reduced in Siponimod-treated mice (IBA1: 4w Veh mean 78.18 *versus* 4w Sipo 23.23; p = 0.0007; APP: 4w Veh mean 583.00 *versus* 4w Sipo 202.38; p = 0.0007). Beyond, axonal densities, as estimated in silver-stained sections, were comparable across all groups (see Fig. 3d), ruling out that the observed differences in APP^+^ spheroids are due to lower axonal densities (4w Veh 81.94 *versus* 4w Sipo 79.77; p = 0.7546). Beyond, densities of OLIG2^+^ cells were found to be decreased at week four, and this decrease was by trend ameliorated by Siponimod (4w Veh mean 289.50 cells/mm^2^ *versus* 4w Sipo 385.25; p = 0.0869).

At week seven, the densities of IBA1^+^ microglia, APP^+^ axonal spheroids and axonal densities were comparable between vehicle- and Siponimod-treated mice (IBA1: 7w Veh mean 12.48 *versus* 7w Sipo 14.16; p = 0.3969; APP: 7w Veh mean 216.38 *versus* 7w Sipo 182.57; p = 0.2946; silver staining: 7w Veh mean 79.14 *versus* 7w Sipo 72.94; p = 0.3969). In contrast, the densities of OLIG2^+^ cells were significantly higher in Siponimod- versus vehicle-treated mice (7w Veh 387.88 *versus* 7w Sipo 566.57; p = 0.0059; Fig. 3e).

**Fig. 4:**
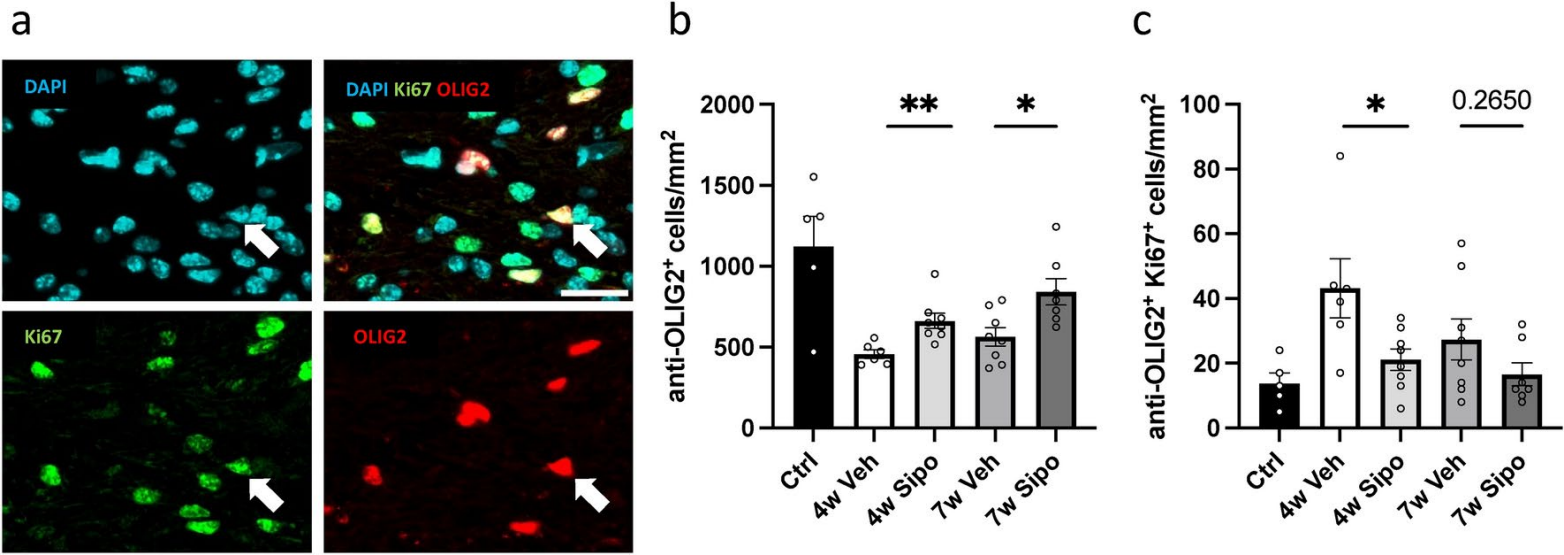
Effects of Siponimod on proliferating oligodendrocyte progenitor cells. (**a**) Representative images of anti-OLIG2/anti-Ki67 immunofluorescence double-stained sections of the midline corpus callosum at the level of the anterior commissure. The white arrow indicates a double-positive cell. Scale bar: 25 μm. (**b**) Quantification of OLIG2^+^ cellular densities and (**c**) quantification of OLIG2^+^/Ki67^+^ double-positive cellular densities (*control* n = 5, *4W Veh* n = 6, *4W Sipo* n = 8, *7W Veh* n = 8, *7W Sipo* n = 7). Statistical analysis: Mann–Whitney test.

The higher densities of OLIG2^+^ cells after week seven in Siponimod- versus vehicle- treated mice might be due to the protection of maturating oligodendrocytes or the induction of OPC proliferation. To analyse this aspect more in detail, sections were processed for anti-OLIG2/Ki67 double immunofluorescence stains to visualize non-proliferating (OLIG2^+^/Ki67^-^) oligodendrocytes and proliferating OPCs (OLIG2^+^/Ki67^+^) (see Fig. 4). In line with our results obtained by permanent immunohistochemical stains (Fig. 3e), densities of all OLIG2^+^ (Fig. 4b) cells were found to be decreased at the end of week four, and this decrease was ameliorated by Siponimod (4w Veh mean 457.50 cells/mm^2^ *versus* 4w Sipo 663.00; p = 0.0013). Furthermore, the densities of all OLIG2^+^ cells at week 7 were significantly higher in Siponimod- compared to vehicle-treated mice (7w Veh 564.63 *versus* 7w Sipo 842.43; p = 0.0151). Regarding the proliferating OPC pool, the densities of OLIG2^+^/Ki-67^+^ cells (Fig. 4c) increased in 4w Veh compared to controls, and this increase was ameliorated by Siponimod-treatment (4w Veh mean 43.17 cells/mm^2^ *versus* 4w Sipo 21.13; p = 0.02). At week 7, the densities of proliferating OPCs were just by trend reduced in Siponimod- versus vehicle-treated mice (7w Veh 27.38 *versus* 7w Sipo 16.57; p = 0.265), suggesting that Siponimod protects maturating oligodendrocytes rather than inducing OPC.

## Discussion

In this study, we showed that Siponimod modestly improved myelination levels during a continuous Cuprizone intoxication challenge, as evidenced by selective recovery of myelin related protein expression levels and a just moderate recovery of OLIG2^+^ cell densities. Notably, Siponimod did not induce OPC proliferation, suggesting its protective effects on differentiating oligodendrocytes.

During a continuous cuprizone challenge over a period of 5 weeks several distinct cellular events occur. First, cells of the oligodendrocyte lineage die, closely paralleled by microglia and astrocyte activation. Oligodendrocyte death is paralleled by disintegration of the myelin sheaths^34^ which results in myelin debris digestion by microglia and, in consequence, demyelination. Although there is rapid regeneration of the oligodendrocyte population following an acute lesion, most of these newly regenerated cells undergo apoptosis if mice remain on a cuprizone diet^35^. We here wanted to know, whether Siponimod can rescue these newly formed oligodendrocytes and thus supports remyelination in the non-supportive environment. We recently investigated whether Siponimod can protect against cuprizone-induced demyelination. In this study, mice were intoxicated with cuprizone for three weeks and myelination levels were analysed either directly after cessation of the cuprizone intoxication or after two weeks of autonomous lesion progression. Under both experimental paradigms, Siponimod profoundly ameliorated the cuprizone-induced pathological changes^21^. Siponimod ameliorated LFB/PAS and anti-PLP staining intensity loss as well as the accumulation of GFAP^+^ astrocytes, IBA1^+^ microglia and APP^+^ spheroids. In contrast, in the current study protective effects were minor. The accumulation of IBA1^+^ microglia as well as APP^+^ axonal spheroids were not different at week 7, and from the four stains used to estimate myelination levels, just two out of four showed statistically significant differences (anti-MBP and anti-MAG). The discrepancy of these results is currently not yet clear. In our initial study, we showed that protective Siponimod effects are mediated via the sphingosine-1-phosphate receptor 5^21^. It might well be that differentiating oligodendrocytes not yet express relevant amounts of this sphingosine-1-phosphate receptor and, thus, cannot induce Siponimod-mediated protective signalling cascades. Further studies are needed to address this important aspect. Nevertheless, some protection was evident, and expression levels of PLP as well as LFB staining intensities recovered by trend.

Several studies have shown that modulation of the sphingosine-1-phosphate signalling cascade can promote remyelination under various experimental conditions^36^. Miron and colleagues demonstrated that Fingolimod (FTY720), which interacts with most of the five sphingosine-1-phosphate receptors (S1PR), enhanced remyelination after lysolecithin-induced demyelination in organotypic cerebellar slice cultures^37^. In particular, Fingolimod increased the number and process extension of oligodendrocytes and OPCs, as well as the numbers of microglia and astrocytes. Comparably, Sheridan and colleagues have shown that Fingolimod ameliorates demyelination in the same model^38^. In this study, during ongoing demyelination (id est, at week four) we found significantly increased densities of IBA1^+^ microglia, which was ameliorated by Siponimod treatment. Since Siponimod attenuates Cuprizone-induced demyelination, and the extent of demyelination positively correlates with the extent of microglial activation in this model, this result was to be expected. A reduction in microglia density by the seven-week time point was also to be expected, as at this advanced stage, the myelin sheaths have largely been phagocytosed, thereby removing a significant trigger factor for microglia activation^39–42^. However, in contrast to the observation of Miron and colleagues, at week seven we did not find any difference of microglia densities between vehicle and Siponimod-treated mice, possibly indicating that the moderate protective effects of Siponimod are not mediated by modulating microglia. Nevertheless, Siponimod might modulate microglia without affecting their densities, an aspect which needs more investigations^43^.

In another study, Pritchard and colleagues showed that splenocytes from MOG-immunized mice or 2D2 transgenic mice could induce demyelination in the cerebellar slice cultures. Treating the splenocytes with Fingolimod, either in vivo or in vitro, prevented this demyelination. Further analysis showed that Fingolimod reduced the release of pro-inflammatory cytokines like IFNγ and IL-6 from the splenocytes^44^. Considering the results of both studies together, it becomes clear that Fingolimod can positively influence both the peripheral aspect of MS pathology, namely autoimmunity, and the central nervous aspect, including neuroinflammation and myelin de- and regeneration.

Similarly to Fingolimod, Siponimod has central and peripheral effects. Within the lymph node, S1P levels are relatively low, creating a gradient with higher S1P concentrations in the efferent lymphatic vessel and the blood. This gradient is critical for guiding lymphocytes out of the lymph node. S1P binds to S1PR1 receptors on lymphocytes, triggering signalling pathways that promote their exit from the lymph node into the efferent lymphatic vessels, facilitating their circulation and subsequent immune surveillance^45–47^. Due to its S1P1 blocking effects, Siponimod exerts immunosuppressive functions in the periphery. In the experimental autoimmune encephalomyelitis-optic neuritis mouse model, Siponimod treatment, both prophylactically and therapeutically, attenuated clinical scores, reduced retinal degeneration, and improved visual function, which was associated with decreased inflammatory infiltrates, demyelination, and a shift in microglial differentiation towards a pro-myelinating phenotype^48^. Regarding central effects, in a Xenopus laevis transgenic model Siponimod treatment improved remyelination in a bell-shaped dose–response manner, and this effect was found to involve the S1PR5^49^. In the Cuprizone model, Siponimod accelerated endogenous remyelination, paralleled by superior outcomes of functional measurements in Siponimod-treated groups^50^. Similar observations have been reported by Dietrich and colleagues^48^ using MRI as a method to evaluate myelination levels. All these findings are in line with our observations, however, in contrast to the above-mentioned studies, we were able to demonstrate that Siponimod can as well activate regenerative pathways in the non-supportive environment. During a continuous Cuprizone exposure, remyelination is unsuccessful because oligodendrocytes in the process of differentiation become susceptible to the Cuprizone toxin. In vitro studies indicate that Cuprizone is specifically toxic to mature oligodendrocytes, while OPCs remain unaffected^51^. Moreover, microglia, astrocytes, and neuronal SH-SY5Y cells exhibit resistance to Cuprizone^51,52^. We believe that OPCs, upon reaching a certain stage of differentiation, become susceptible to Cuprizone and perish, leading to remyelination failure during prolonged intoxication. However, if Cuprizone is eliminated from the diet, OPCs can fully differentiate and repair the myelin in the brain’s affected white and grey matter. Supporting this concept, it has been shown that Cuprizone hinders the differentiation of oligodendrocytes in vitro^53^.

To determine whether newly formed oligodendrocytes are protected by Siponimod, it was crucial to begin the treatment at a point where OPC differentiation had already commenced in the experimental mice. As previously published by our group, low numbers of proliferating OLIG2^+^ cells can be found in the control group and the mice intoxicated with cuprizone for 1 week. In contrast, significantly higher numbers were seen at weeks 3 and 5. Additionally, quantifying CC1^+^ cells showed significantly reduced numbers at weeks 1 and 3 but a rapid increase by the fifth week of Cuprizone treatment despite ongoing intoxication^30^. These findings indicate that remyelination, involving OPC proliferation and maturation, is an active process between weeks 3 and 5 in this model^25^.

As previously outlined by our group, some important aspects should be considered when using the Cuprizone model^16^. Potential protective drug effects are best studied using an experimental setting where damage is semi-maximal. In the rostral part of the corpus callosum, the demyelination along the midline is patchy and incomplete. In contrast, at more caudal levels in the body of the corpus callosum, demyelination of its midline is severe and almost complete. During this study, we analysed not only the rostrum but also the body of the corpus callosum and found after four weeks of Cuprizone intoxication, severe demyelination, paralleled by intense microgliosis spreading into the surrounding hippocampus formation. Indeed, just minor remyelination-supporting Siponimod effects have been observed at this brain level. We assume that the intense neuroinflammation in this region prevented the protective effects of Siponimod from taking effect.

In summary, this study makes another relevant contribution to understand the mechanism of action of S1PR modulators in general, and Siponimod in particular. S1PR modulators are a promising class of substances for the treatment of MS, as well as other neurodegenerative diseases. However, many questions remain unanswered. First, which cells express the various S1PRs in the brain, and how does their expression pattern change in the diseased state? The development of highly specific antibodies would be necessary to answer this critical question. Second, do the various S1PR modulators act as functional antagonists at all receptors? At least for Siponimod, we have initial findings from cell culture studies indicating that while Siponimod acts as a functional antagonist at S1PR1, it acts as an agonist at S1PR5. Furthermore, to maximise drug safety and efficacy, additional in vitro and in vivo studies must follow to demonstrate the effects of selective S1P modulators that bind only to one of the five receptor subtypes in relevant animal models.

## Data Availability

The data that support the findings of this study are available from the corresponding author upon reasonable request.

## Abbreviations

CC: Corpus callosum
CNS: Central nervous system
Ctrl: Control
d: Day(s)
DAPI: 4',6-Diamidino-2-phenylindole
h: Hour(s)
H_2_O_2_: Hydrogen peroxide
IBA1: Ionized calcium-binding adapter molecule 1
i.e.: *id est*
IFNγ: Interferon- γ
IL-6: Interleukin-6
LFB/PAS: Luxol fast blue/periodic acid-schiff
mCC: Medial corpus callosum
min: Minute(s)
MS: Multiple sclerosis
MRI: Magnetic resonance imaging
OPC: Oligodendrocyte progenitor cell
PBS: Phosphate-buffered saline
PFA: Paraformaldehyde solution
PLP: Myelin proteolipid protein
PPMS: Primary-progressive MS
ROI: Region of interest
RRMS: Relapsing-remitting MS
Sipo: Siponimod
SPMS: Secondary progressive MS
S1P: Sphingosine-1 phosphate
S1PR: Sphingosine-1 phosphate receptor
Tris/EDTA: Tris(hydroxymethyl)aminomethane/ethylenediaminetetraacetic acid
Veh: Vehicle
w: Week(s)
